# Economic Evaluation of Rituximab + Recombinant Human Thrombopoietin vs. Rituximab for the Treatment of Second-Line Idiopathic Thrombocytopenic Purpura in China

**DOI:** 10.3389/fmed.2021.657539

**Published:** 2021-03-18

**Authors:** Mingjun Rui, Yingcheng Wang, Zhengyang Fei, Ye Shang, Aixia Ma, Hongchao Li

**Affiliations:** ^1^School of International Pharmaceutical Business, China Pharmaceutical University, Nanjing, China; ^2^Center for Pharmacoeconomics and Outcomes Research, China Pharmaceutical University, Nanjing, China

**Keywords:** cost-effectiveness (CE), immunologic thrombocytopenic purpura, rituximab, RhTPO, Markov model

## Abstract

**Objective:** This study aimed to compare the economic evaluation of recombinant human thrombopoietin+rituximab (rhTPO + RTX) vs. RTX as second-line treatment for adult patients with immunologic thrombocytopenic purpura in China.

**Methods:** The Markov model was used in our research. The response rate and relapse rate data were derived from two clinical trials and one retrospective study. Cost and utility values were derived from published literature, a third-party database, and healthcare documents. In addition, one-way sensitivity analysis and probabilistic sensitivity analysis were performed to observe the stability of the model and data source.

**Results:** In the Markov model, compared with RTX, rhTPO+RTX yielded an additional 0.04 QALYs, with an incremental cost of 2,802 USD. The ICER was 69,097 USD/QALY. According to the results from the one-way sensitivity analysis, complete response of rhTPO+RTX, utility of complete response and response of RTX were the main drivers in the model. The results from the probabilistic sensitivity analysis demonstrated that there was a 100% probability that rhTPO+RTX was not cost-effective vs. RTX alone at a threshold of $10,805/QALY and an 84% probability at a threshold of $32,415/QALY.

**Conclusion:** RTX+rhTPO was not more cost-effective than RTX alone as second-line treatment for adult patients with immunologic thrombocytopenic purpura in China.

## Introduction

Idiopathic thrombocytopenic purpura (ITP), also referred to as primary immune thrombocytopenia, is a disorder mediated by autoimmunity that mainly leads to the increased destruction and decreased production of platelets (1). The disease is characterized by lower peripheral platelet counts than normal. In adults, ITP is a chronic disease with a very low rate of spontaneous remission, requiring drug intervention (2). Epidemiological research on ITP in China shows that the incidence of ITP is ~9.5 in 100,000 per year, and its incidence in adult females is greater than that in adult males (2:1 ratio), which might be related to the fact that adult women are more likely to develop autoimmune system diseases (3). Chronic ITP causes a certain socioeconomic burden and decreases patients' quality of life due to the occurrence of bleeding and the increase in mortality (4). Approximately 8.7% of patients have another autoimmune system disease, bleeding or thrombosis. The death risk of ITP patients is 1.5 times higher than that of the general population (5). Various studies have shown that adult ITP patients incur significant medical costs (the costs are mainly driven by the costs of hospitalization, bleeding, medication, and surgery) and have lower productivity at work. From the global perspective, the economic burden of chronic ITP at present has been illustrated. In Europe, the annual hospitalization cost was ~€ 26,581 per patient in 2007, and the cost of emergency medicine for bleeding caused by ITP was also high in France (6). In the US, the annual cost of drug treatment alone for ITP has been estimated to be ~$28,000 on average for each patient per year (2000−2003) (7). Positive treatment was recommended for adult ITP patients with platelet counts lower than 30 × 10^9^ or between 30 and 50 × 10^9^ and with bleeding or bleeding risk based on the current evidence (8).

In China, glucocorticoids and intravenous immunoglobulin G are usually used as first-line treatment for adult ITP patients (9). However, the side effects of the long-term use of glucocorticoids need attention. Second-line treatment includes rituximab (RTX), recombinant human thrombopoietin (rhTPO), rhTPO+RTX, and splenectomy. The study of Zhou et al. (10) found that RTX combined with rhTPO as second-line treatment had high benefits in terms of the remission rate and the control of the recurrence rate, so this combination has been recommended by the Chinese guideline on the diagnosis and management of adult primary immune thrombocytopenia (version 2020) (9). However, compared with RTX monotherapy, the cost of combination therapy is relatively high, and there is no related economic evaluation in China. The purpose of the study was to evaluate the cost-effectiveness of rhTPO+RTX as second-line treatment in patients with chronic ITP in China.

## Methods

### Patient Population and Model Structure

**Figure 1:**
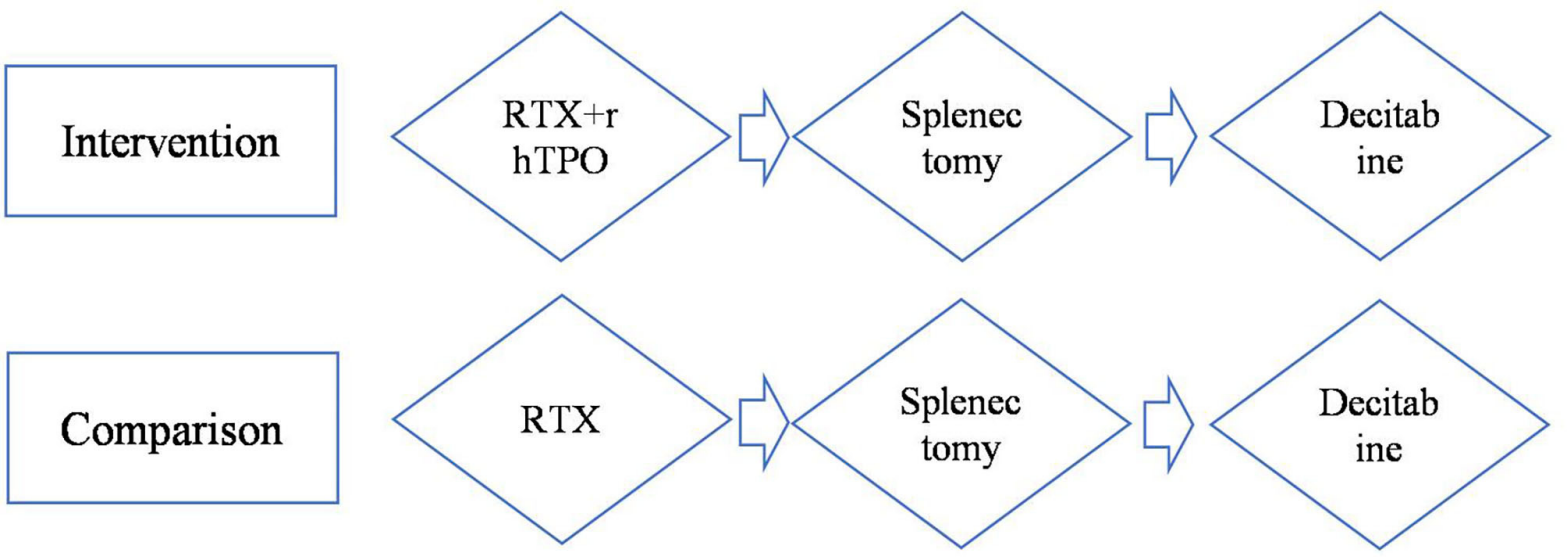
Model treatment pathways for adult immune thrombocytopenia. RTX, rituximab; rhTPO+RTX, recombinant human thrombopoietin+rituximab.

A treatment-sequence Markov model was developed to assess the cost-effectiveness of rhTPO+RTX vs. RTX as second-line treatment for adult chronic ITP patients who had failed to respond to corticosteroids or who had experienced relapse. The patient cohort was defined as having similar characteristics as patients from the rhTPO+RTX phase 3 trial (10). All of the patients were assumed to be non-splenectomized, and the median age was assumed to be 42 years. The following two treatment strategies were compared (Figure 1):

Since decitabine is recommended as third-line treatment for adult chronic ITP patients (9), it was assumed that decitabine was received after splenectomy in this pathway. After failing RTX/rhTPO+RTX, patients moved on to splenectomy followed by decitabine.

A Markov model with an embedded decision tree was used with a month cycle, and a 30-year time horizon was taken in the base-case analysis, developed from the perspective of the Chinese health care system. A 30-year time horizon was thought to be appropriate, since adult ITP tends to be a chronic disease and the median age of the patients was 42; a 1-month cycle was used to match the evaluation schedule used in the clinical trial of rhTPO+RTX/RTX, splenectomy, and decitabine. Costs and outcomes were discounted at a 5% annual rate, as recommended by the China Guidelines for Pharmacoeconomic Evaluations (11). The treatment sequence used in the model was based on the Chinese guidelines on the diagnosis and management of adult primary immune thrombocytopenia (version 2020) (9).

**Figure 2:**
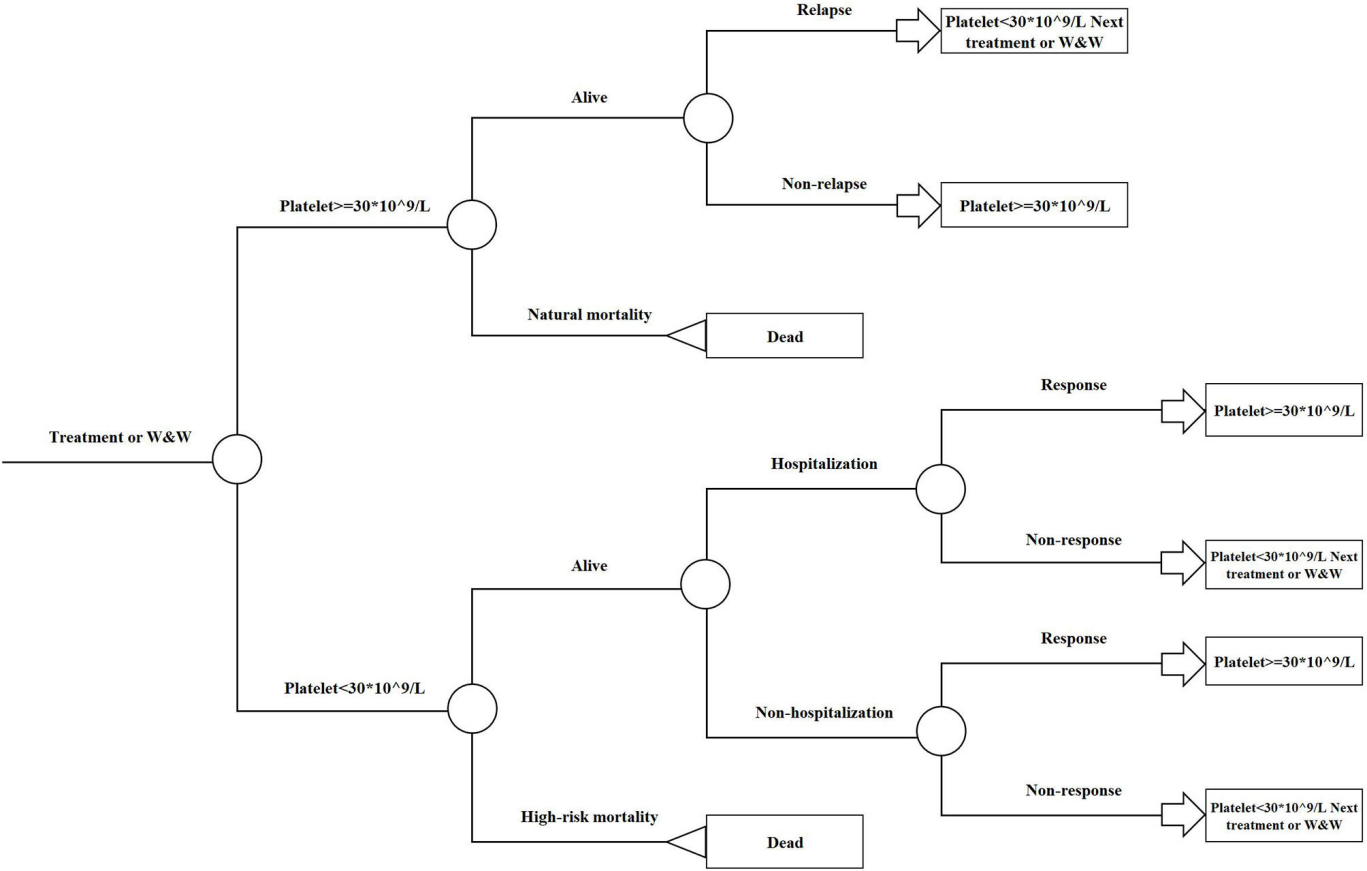
Embedded decision tree overview. W&W, Watch-and-wait strategy.

**Figure 3:**
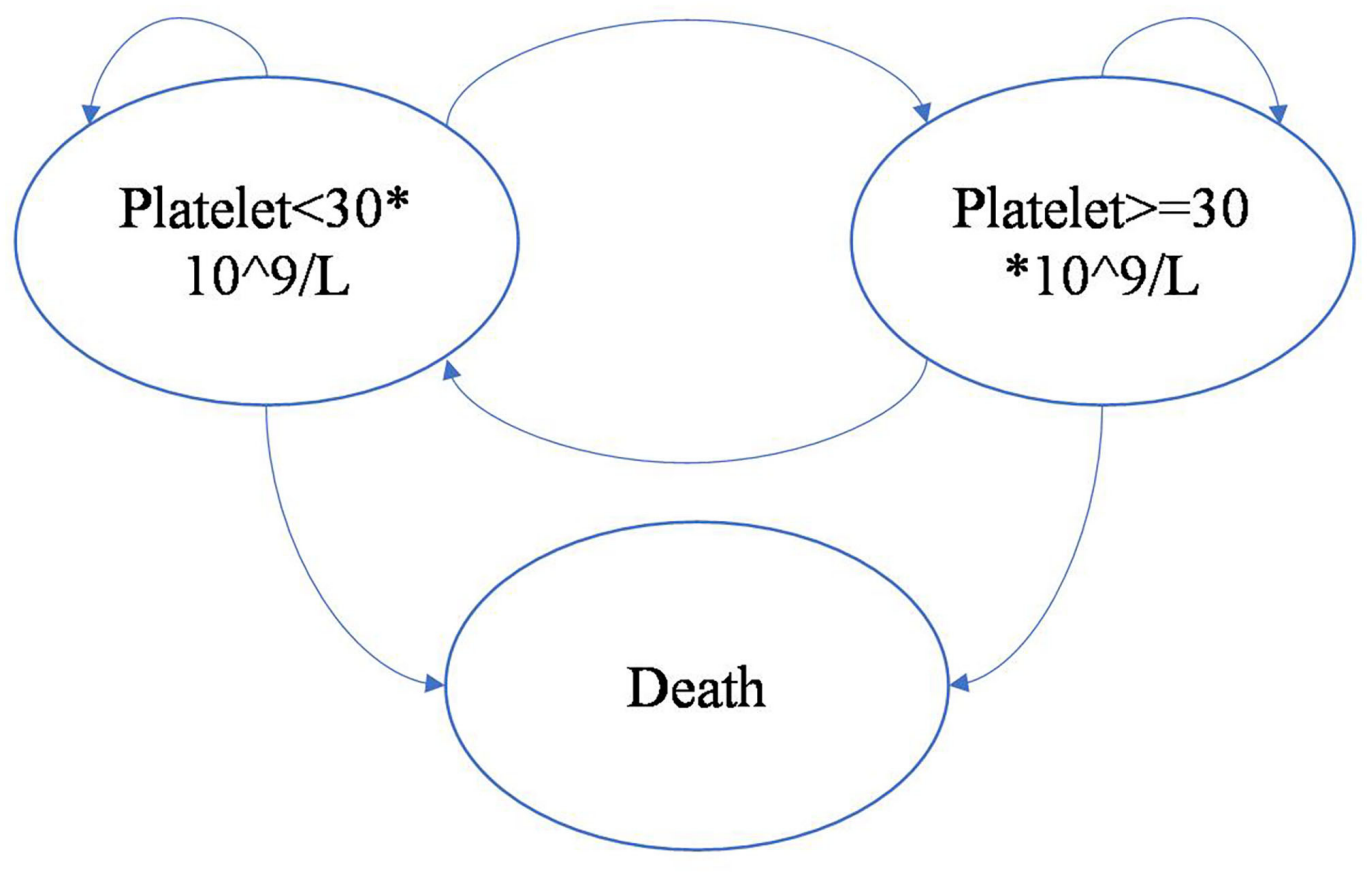
Markov model overview driven by platelet response.

The model was driven by platelet response (platelet count ≥30 × 10^9^/L), which determined effectiveness and progression along the treatment pathway (Figures 2, 3). The patients started on the first treatment in the pathway and progressed to the next treatment or were managed with the watch-and-wait strategy if they did not have a complete response (CR) or response (R) or overall response (OR = CR+R) if they experienced relapse (platelet count <30 × 10^9^/L) after responding. Each relapse on active treatment was followed by one cycle period of the watch-and-wait state before initiating the next active treatment.

### Clinical Inputs

Clinical efficacy data were derived from three clinical trials. The efficacy of each treatment within the model was characterized by the following parameters:

- Probability of CR and R with rhTPO+RTX/RTX treatment;
- Probability of OR with splenectomy treatment and decitabine treatment;
- Time to relapse with platelet count <30 ×10^9^/L of patients with an initial response (including CR, R, and OR) to rhTPO+RTX/RTX treatment, splenectomy treatment, and decitabine treatment.

Efficacy data for rhTPO+RTX/RTX were taken from an open-label prospective randomized controlled clinical trial in 12 centers in China reported by Zhou et al. (10). This study compared the efficacy and safety of RTX plus rhTPO with RTX alone in patients with ITP who had failed to respond to corticosteroids or who had experienced relapse. The patients were randomized at a ratio of 2:1 into 2 groups: the combination group (rhTPO+RTX, *n* = 77) and the monotherapy group (RTX, *n* = 38). The probability of CR and R in the rhTPO+RTX/RTX treatment group was also considered in the Markov model because the study found that the combination of RTX and rhTPO could significantly increase the CR rate, which might bring more benefit to patients.

Efficacy data for splenectomy treatment were taken from one retrospective review of the experience at Institute of Hematology & Blood Diseases Hospital between 1990 and 2003 with adults with chronic ITP (12). Data from 149 chronic ITP patients were retrospectively analyzed, and relapse-free survival was estimated by Kaplan–Meier analysis. In addition, data for decitabine treatment was taken from a prospective, multicenter study that evaluated the efficacy and safety of low-dose decitabine in adult patients with refractory immune thrombocytopenia by Zhou et al. (13).

The time to relapse in rhTPO+RTX/RTX treatment was estimated by fitting parametric survival distributions to pseudo-individual participant data (IPD) derived from digitized Kaplan-Meier curves from the study by Zhou et al. (13), and pseudo-IPD data were extracted with Engauge Digitizer software. We considered five standard parametric models to fit two curves: exponential distribution, Weibull distribution, Gompertz distribution, loglogistic distribution, and lognormal distribution. The lognormal distribution was the best-fitting curve according to the Akaike information criterion (AIC) (Supplementary Material). The exponential distribution was used to fit the time to relapse in splenectomy treatment and decitabine treatment because its constant risk was suitable for the memoryless characteristics of the Markov model. Although it is important to consider adverse events in the treatment of ITP, the incidence of events was not clearly explained in the literature, and the available evidence of its impact on cost and quality of life was limited. Therefore, the cost and disutilities of adverse events were not included in the model.

### Hospitalization Events

In the study by Portielje et al. (5), the incidence of hospital admissions was assessed. The study showed that for those who did not respond to the treatment, the incidence of ITP-related admissions per 1,000 person-years was 183. The per-cycle probability of the incidence was calculated as 0.0167.

### Mortality

The model included both natural mortality and high-risk mortality. When platelet count ≥30 ×10^9^/L, natural mortality derived from the Chinese life tables was used, and when platelet count <30 ×10^9^/L, high-risk mortality, which was assumed to be 1.5 ^*^ natural mortality according to the study by Portielje et al., was used (5).

### Utility

Due to the lack of utility data for Chinese adult ITP patients, utilities from a time trade-off (TTO) survey conducted in the UK were included in our base-case analysis (14). TTO analysis was used in that survey to directly measure the utility values for 359 adult patients with ITP of the general public, and some assumptions were made as follows:

- The utility when platelet count ≥30 ×10^9^/L was assumed to be the same as sufficient platelets (≥50 * 10^9^/L) with no outpatient bleed state in TTO analysis;
- The utility when platelet count <30 ×10^9^/L was assumed to be the same as low platelets (<50 * 10^9^/L) with no outpatient bleed state in TTO analysis.

### Costs and Resource Use

Costs were assessed in dollars, with 2020 values, from the perspective of the Chinese health care system. The drug acquisition and costs associated with splenectomy treatment and hospitalization events were considered in the model. The resource use and costs were derived from a third-party database (www.yaozhi.com) and published literature (15, 16) in China.

All patients were assumed to weigh 65 kg (17) when estimating the dosages of decitabine treatment, and there was no dose sharing between patients according to the situation in China. The clinical inputs, utilities and costs used in the model are shown in Table 1.

**Table 1:** I Key inputs for the Markov model.

	**Deterministic**	**Distribution**	**Low**	**High**	**References**
**QoL utility (per year)**					
CR utility	0.86	Beta	0.78	0.95	(14)
R utility	0.84	Beta	0.76	0.86	(14)
NR utility	0.54	Beta	0.49	0.59	(14)
**Clinical inputs**					
CR of rhTPO+RTX	0.45	Beta	0.36	0.54	(10)
R of rhTPO+RTX	0.34	Beta	0.27	0.41	(10)
CR of RTX alone	0.24	Beta	0.19	0.28	(10)
R of RTX alone	0.47	Beta	0.38	0.57	(10)
OR of splenectomy treatment	0.83	Beta	0.66	0.99	(12)
OR of decitabine treatment	0.51	Beta	0.41	0.61	(13)
Hospitalization	0.18	Constant			(5)
**Direct costs per cycle ($)**					
Cost of rhTPO	2,883	Gamma	2,860	3,016	YAOZHI
Cost of RTX	1,021	Gamma	628	1,474	YAOZHI
Cost of splenectomy treatment	1,545	Gamma	1,171	1,545	(15)
Cost of decitabine treatment	313	Gamma	226	618	YAOZHI
Cost of hospitalization	3,294	Gamma	1,647	4,940	(16)
Cost of administration	1.4	Gamma	1	2	Health document
Discount	0.05	Constant	0	0.08	(11)

*RTX, rituximab; rhTPO+RTX, recombinant human thrombopoietin+rituximab; CR, complete response; R, response; NR, non-response; OR, overall response*.

### Model Outputs

The total costs and quality-adjusted life years (QALYs) per patient and the incremental cost-effectiveness ratio (ICER) were calculated.

## Sensitivity Analysis

### One-Way Sensitivity Analysis

One-way sensitivity analysis was performed to identify the parameters to which the model was most sensitive. The upper and lower limits of the inputs were defined by 95% confidence intervals where possible and derived from the original literature or with plausible variation around the base-case values by 20%.

### Probabilistic Sensitivity Analysis

Probabilistic sensitivity analysis (PSA) was conducted using 1,000 iterations to examine parameter uncertainty over the entire model, and cost-effectiveness acceptability curves (CEACs) were then calculated.

### Ethics

The data was based on previously published trial, not from the database or the medical records. We used the Engauge Digitizer software to get the time to relapse information from the figures in the paper and reconstructed data by ourselves, which was explained in the Section “Clinical Inputs.” Besides, the utilities and costs were derived from the published literatures, so ethics approval or specific consent procedures were not required for this study.

## Results

### Base Case

The base-case model results (Table 2) indicated that rhTPO+RTX treatment resulted in an average of $2,802 higher costs for 0.041 QALYs over a 30-year timespan than RTX. In the base-case analysis with a 30-year time horizon, the economic, and health outcomes calculated using the Markov model are presented in Table 2. We found that the ICER in the Markov model was $69,097/QALY, which was higher than the willingness-to-pay thresholds of $10,805/QALY (1 GDP per capita) and $32,415/QALY (3 GDP per capita) in China.

**Table 2:** Base-case cost-effectiveness of rhTPO+RTX compared with RTX.

	**rhTPO+RTX**	**RTX**
Total costs	8,789	5,987
QALYs gained	10.89	10.85
	**rhTPO+RTX vs. RTX**	
Incremental total costs	2,802	
Incremental QALYs gained	0.04	
ICER	69,097	

*RTX, rituximab; r hTPO+RTX, recombinant human thrombopoietin+ rituximab*.

### One-Way Sensitivity Analysis

**Figure 4:**
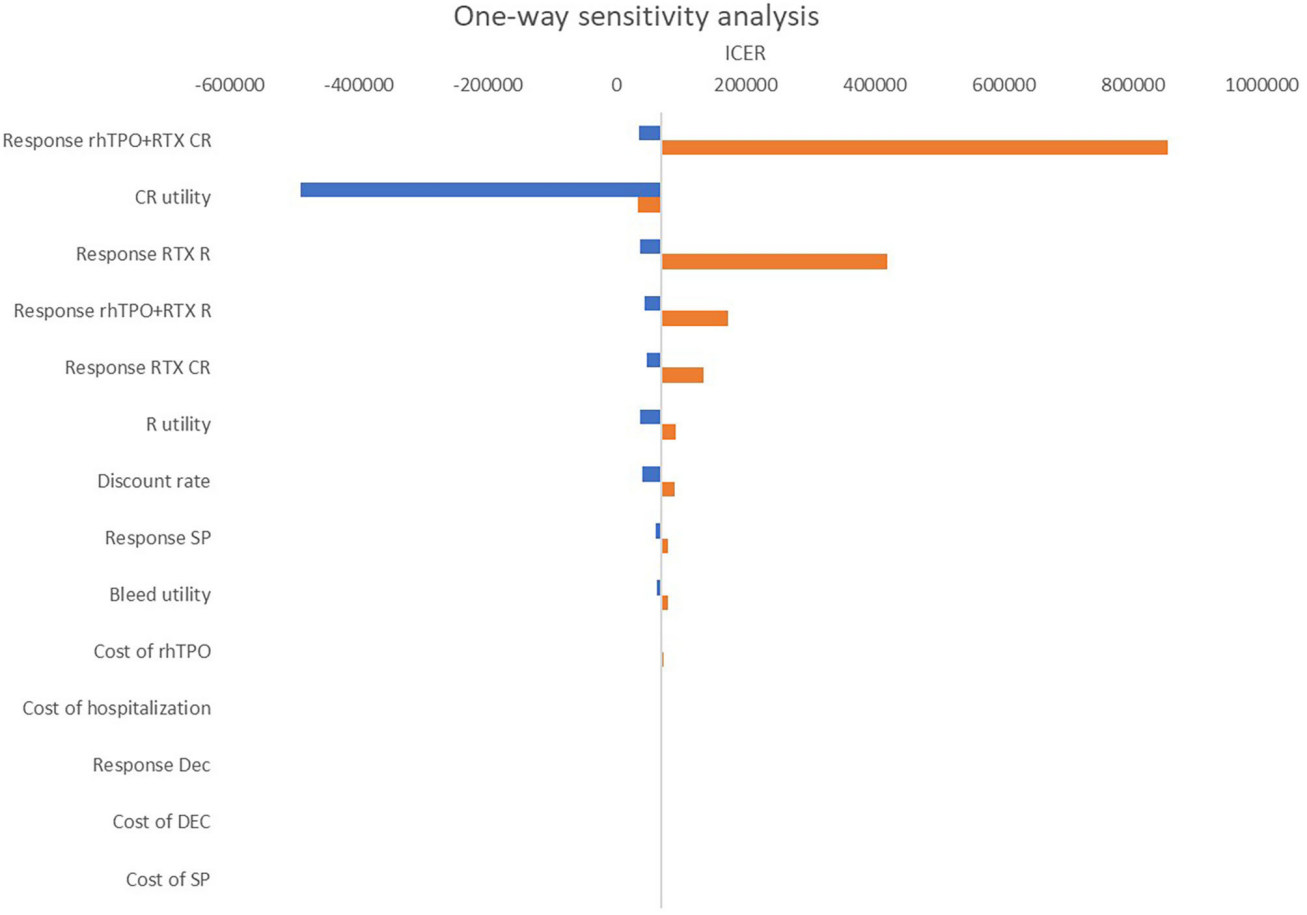
One-way sensitivity analysis: rhTPO+RTX vs. RTX. RTX, rituximab; rhTPO+RTX, recombinant human thrombopoietin+rituximab; CR, complete response; R, response; NR, non-response; Dec, decitabine; SP, splenectomy.

Figure 4 shows the results of the one-way sensitivity analysis. The main driver variables were CR of rhTPO+RTX, utility of CR and R of RTX. It should be noted that changes in these variables might cause the result to be reversed at a threshold of $10,805/QALY ~ $32,415/QALY.

### Probabilistic Sensitivity

**Figure 5:**
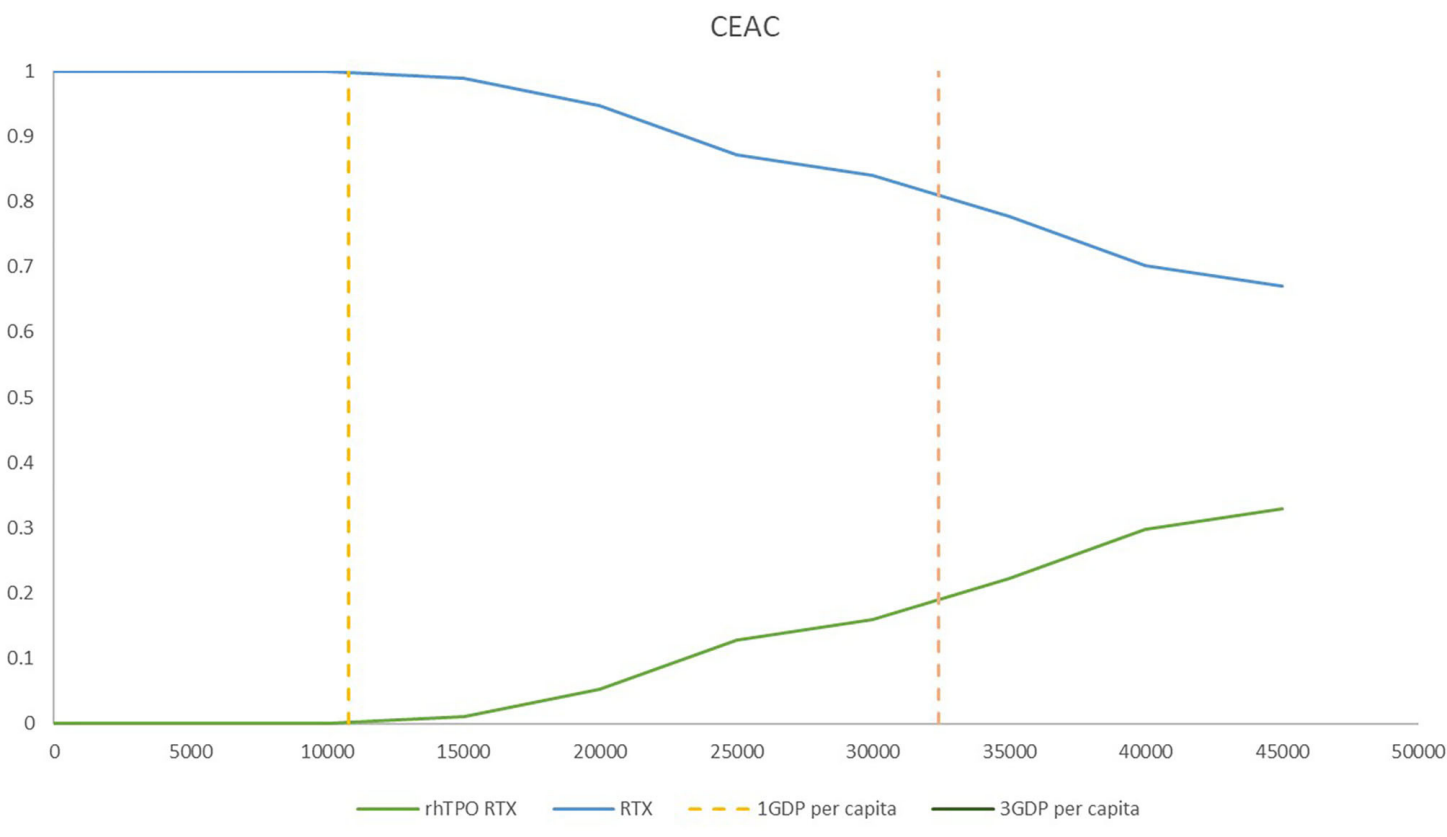
Cost-effectiveness acceptability frontier for rhTPO+RTX and RTX. RTX, rituximab; rhTPO+RTX, recombinant human thrombopoietin+rituximab.

The CEACs at different willingness-to-pay thresholds in the model are shown in Figure 5. The results from PSA showed that treatment with RTX alone was more cost-effective than treatment with rhTPO+RTX in the majority of cases (Figure 5). There was a 100% probability that rhTPO+RTX was not cost-effective vs. RTX alone at a threshold of $10,805/QALY and an 84% probability at a threshold of $32,415/QALY (Figure 5).

## Discussion

According to the base-case results, the total utilities of the rhTPO+RTX group were higher because the probability of CR and overall response in the rhTPO+RTX group was higher. The results of this economic evaluation indicated that rhTPO+RTX treatment was more costly and more effective than RTX alone for the second-line treatment of adult ITP. The estimated ICER was $69,097/QALY in the model, which indicated that rhTPO+RTX treatment was not cost-effective for the second-line treatment of adult ITP in China. According to the results from the one-way sensitivity analysis, CR of rhTPO+RTX, utility of CR and R of RTX were the main drivers in the model. The CEAC showed that there was a 100% probability that rhTPO+RTX was not cost-effective vs. RTX alone at a threshold of $10,805/QALY and an 84% probability at a threshold of $32,415/QALY, which confirmed the conclusion based on the base-case results.

There are many clinical symptoms of primary immune thrombocytopenia, including asymptomatic thrombocytopenia, skin and mucosal hemorrhage, severe visceral hemorrhage, fatal intracranial hemorrhage, etc. The disease will bring a certain loss of quality of life to patients. In China, RTX is approved for (1) relapsed or refractory, low-grade or follicular, CD20-positive B-cell non-Hodgkin's lymphoma as a single agent; (2) previously untreated follicular, CD20-positive B-cell non-Hodgkin's lymphoma; and (3) previously untreated and previously treated CD20-positive chronic lymphocytic leukemia (http://zy.yaozh.com/instruct/20180711sms/142.pdf). Although it has not been approved for the treatment of adult primary immune thrombocytopenia, RTX alone and rhTPO+RTX have been listed in the Chinese guidelines on the diagnosis and management of adult primary immune thrombocytopenia (version 2020) as a clear recommendation (9). Although there is consensus among clinical experts confirming the effectiveness and safety of the two therapies, the question of whether they are cost-effective in clinical application has been unanswered in China. To the best of our knowledge, this study is the first in China to compare the economic evaluation of rhTPO+RTX and rituximab alone for the treatment of adult chronic ITP.

Several limitations also need to be recognized in our study. First, due to the lack of preference-based research on Chinese adult immune thrombocytopenia patients, utility value data for Chinese adult patients with ITP were not available. At the same time, there was not enough evidence to support the utilities obtained by the mapping method, so utilities from the UK TTO study were used for analysis. Second, this article did not consider adverse events in the study. The reasons were as follows: (1) according to clinical inputs, the probabilities of adverse events with these two treatment strategies were relatively low and close, without a significant difference; (2) there was not enough evidence to support the cost of related adverse events and the disutilities of adverse events, which would have a certain impact on the final ICER. Besides, in this article, we assumed that all patients had never received splenectomy treatment, which might be slightly inconsistent with the real-world situation; however, considering that the number of patients who had received splenectomy treatment recorded in clinical inputs was <10%, this would not lead to a great bias. In addition, we could find the overall response of RTX alone in the clinical trial is 71.1%, which is higher than most previously reported OR results (18, 19), which might affect the final results to some extent. The data used in the manuscript was from clinical trials because there was no related information about the efficacy of the rhTPO+RTX in the real-word evidence and data from the same source could reduce the heterogeneity of research. For hospitalization events, difference of the hospitalization rates might exist for two types of treatments (rhTPO+RTX vs. RTX) due to the difference of efficacy and response rates for two treatments. For the higher CR and OR of rhTPO+RTX, the hospitalization rates would be lower in this group, which would lead to the lower ICER. Finally, for the complex real-world situations, generalizability is also a limitation. According to the Chinese guidelines on the diagnosis and management of adult primary immune thrombocytopenia (version 2020), there are other second-line treatment options like eltrombopag, romiplostim, azathioprine, cyclosporine A, danazol, vinca alkaloid etc, which may affect the model treatment pathways and further impact the cost evaluations.

## Conclusion

In summary, the treatment of rhTPO+RTX, compared with rituximab monotherapy, is likely to be not cost effective in China as second-line treatment for adult patients with immunologic thrombocytopenic purpura.

## Data Availability Statement

All datasets generated for this study are included in the article/Supplementary Material.

## Ethics Statement

The data was based on previously published trial, not from the database or the medical records. We used the Engauge Digitizer software to get the time to relapse information from the figures in the paper and reconstructed data by ourselves, which was explained in the Section Clinical Inputs. Besides, the utilities and costs were derived from the published literatures, so ethics approval or specific consent procedures were not required for this study.

## Author Contributions

MR, YW, and HL developed the economic model and performed the analyses. MR, ZF, and YS interpreted the results and wrote the draft manuscript. MR, YW, ZF, HL, and AM reviewed, analyzed, and interpreted the data. MR and HL contributed to the design of the primary model and the interpretation of the results. All authors reviewed and approved the final version.

## Conflict of Interest

The authors declare that the research was conducted in the absence of any commercial or financial relationships that could be construed as a potential conflict of interest.
