# Supplementary material for: Economic Evaluation of Rituximab + Recombinant Human Thrombopoietin vs. Rituximab for the Treatment of Second-Line Idiopathic Thrombocytopenic Purpura in China
Source: Front Med (Lausanne). 2021 Mar 18;8:657539. doi: 10.3389/fmed.2021.657539 (PMC8012846; doi:10.3389/fmed.2021.657539)
Supplement: Supplementary file 1 [file Data_Sheet_1.docx]

# Supplementary Material

Economic Evaluation of Rituximab+rhTPO versus Rituximab for the Treatment of Second-line ITP in China

# Estimation of Parametric Survival Distributions for Time to relapse

Table S1 Results of fitting to the observed data

|  |  | Exponential | Weibull | Gompertz | Log-logistic | Log normal |
| --- | --- | --- | --- | --- | --- | --- |
| TTR RTX+rhTPO | AIC | 112.38 | 114.09 | 111.78 | 111.60 | 110.09 |
| TTR RTX | AIC | 256.18 | 255.51 | 244.15 | 244.08 | 240.94 |

Table S2 Best fitting and the value of the parameter

|  | Fitting | λ/μ | σ |
| --- | --- | --- | --- |
| TTR RTX+rhTPO | Lognormal(μ, σ) | 2.68 | 1.46 |
| TTR RTX | Lognormal(μ, σ) | 2.09 | 1.34 |
| TTR SP | Exponential (λ) | -5.57 | / |
| TTR Dec | Exponential (λ) | -3.27 | / |
